# Forever in the dark: the cave-dwelling azooxanthellate reef coral *Leptoseris troglodyta* sp. n. (Scleractinia, Agariciidae)

**DOI:** 10.3897/zookeys.228.3798

**Published:** 2012-10-11

**Authors:** Bert W. Hoeksema

**Affiliations:** 1 Department of Marine Zoology, Naturalis Biodiversity Center, P.O. Box 9517, 2300 RA Leiden, The Netherlands

**Keywords:** Cavernicolous, colonial, dwarfism, extramural budding, monocentric, skiophilous, solitary, troglobiotic

## Abstract

The coral species *Leptoseris troglodyta* **sp. n.** (Scleractinia, Agariciidae) is described as new to science. It is the first known azooxanthellate shallow-water agariciid and is recorded from the ceilings of caves at 5-35 m depth in West Pacific coral reefs. The corals have monocentric cup-shaped calices. They may become colonial through extramural budding from the basal coenosteum, which may cause adjacent calices to fuse. The size, shape and habitat of *Leptoseris troglodyta* are unique compared to other *Leptoseris* species, many of which have been recorded from mesophotic depths. The absence of zooxanthellae indicates that it may survive well in darkness, but endolithic algae in some corals indicate that they may be able to get some light. The presence of menianes on the septal sides, which may help to absorb light at greater depths in zooxanthellate corals, have no obvious adaptive relevance in the new species and could have been inherited from ancestral species that perhaps were zooxanthellate. The new species may be azooxanthellate as derived through the loss of zooxanthellae, which would be a reversal in *Leptoseris* phylogeny.

## Introduction

Reef-dwelling species of the genus *Leptoseris* Milne-Edwards and Haime, 1849 (Scleractinia: Agariciidae) consist of foliaceous corals that are common in poorly illuminated environments, such as the deepest parts of reef slopes and vertical rocky walls with crevices, caves, tunnels and overhangs (Dinesen 1980, 1982, 1983; Waheed and Hoeksema in press). They are considered skiophilous (shade-loving) or cavernicolous, i.e., living in caves (Dinesen 1982, 1983). Because they appear to show more preference for dark habitats than many other reef corals, *Leptoseris* species constitute an important component of zooxanthellate scleractinian coral communities at mesophotic depths (30–150 m) (Kahng and Kelley 2007, Chan et al. 2009, Bare et al. 2010, Bongaerts et al. 2010, Kahng et al. 2010, Rooney et al. 2010, Dinesen et al. 2012). They may even occur deeper, with records of 153 and 165 m by *Leptoseris hawaiiensis* Vaughan, 1907, in the Pacific Ocean (Maragos and Jokiel 1986, Kahng and Maragos 2006), and 145 m by *Leptoseris fragilis* Milne Edwards and Haime 1849, in the Red Sea (Fricke and Schuhmacher 1983, Fricke et al. 1987, Kaiser et al. 1993).

Because some *Leptoseris* species inhabit deep and poorly accessible habitats, they may not all be well known. An example is the recently discovered *Leptoseris kalayaanensis* Licuanan & Aliño, 2009, which so far has only been recorded from rocky substrates at 13–28 m depth in the South China Sea basin (Licuanan and Aliño 2009, Hoeksema et al. 2010). It shows a distinct brown and white coloured pattern on its upper surface, consisting of areas that are either with or without zooxanthellae.

The Agariciidae were not known to include true deep-sea species but according to recent phylogenetic studies, the solitary attached deep-water coral *Dactylotrochus cervicornis* (Moseley, 1881), which was originally classified with the Caryophylliidae, is also a member of the Agariciidae (Kitahara et al. 2010, 2012). This species has a recorded depth range of 73–852 m, is therefore considered ahermatypic and probably azooxanthellate (for terminology see Schuhmacher and Zibrowius 1985, Cairns and Kitahara 2012). It is monocentric and has smooth-edged septa that bear 2–5 elongate ridges (menianes or latera), which are considered characteristic for the Agariciidae (Kitahara et al. 2010, 2012).

Because *Dactylotrochus cervicornis* is predomintly from deep water, it is considered the first known extant azooxanthellate agariciid. *Dactylotrochus cervicornis* holds a basal position in a recent phylogeny reconstruction of extant Agariciidae and because extinct solitary agariciids from the Middle Cretaceous were also solitary, it is assumed that the ancestor of the Agariciidae, which nowadays predominantly consist of colonial and zooxanthellate species, was also solitary and azooxanthellate (Kitahara et al. 2012).

In the present paper a new agariciid coral species is described that is entirely azooxanthellate and dwells on ceilings of caves in steep reef slopes and walls. No co-occurrence with any zooxanthellate scleractinians was observed. Although its calices are relatively small, cup-shaped and predominantly monocentric, it resembles species of *Leptoseris*,which otherwise is known to consist of zooxanthellate species with polycentric (“circumoral”) calices (Wells 1956). It is furthermore unique among extant reef-dwelling Agariciidae because it is modular (colonial) through extramural budding by growing new calices from a basal coenosteum, which eventually may fuse. It has been found in the western Pacific, including eastern Indonesia, central Philippines, Papua New Guinea, Palau and Guam. Most of its presently known distribution range overlaps with the centre of maximum marine species richness, the so-called Coral Triangle (Hoeksema 2007).

## Methods

Specimens were observed, photographed and collected while diving with the help of SCUBA. All specimens were encountered below 5 m depth on the ceilings of caves inside steep reef slopes and walls, usually in areas with limestone outcrops. The caves measured one to several meters in width and several meters in length, enabling easy access and maneuvering for observations. Use of an underwater torch was indispensable to locate the corals. Collected specimens were soaked in fresh water or in sodium hypochlorite solution for cleaning. They were deposited in the Coelenterata collection (RMNH Coel.) of Naturalis Biodiversity Center, Leiden (formerly known as Rijksmuseum van Natuurlijke Historie). Specimens from Cebu were already available in the RMNH collection before they were photographed and collected for the present research. SEM photographs were taken with a Jeol 6480LV electron microscope operated at 10 kV.

## Systematic section

### Order Scleractinia Bourne, 1905. Family Agariciidae Gray, 1847. Genus *Leptoseris* Milne Edwards & Haime, 1849

#### Leptoseris troglodyta sp. n.

urn:lsid:zoobank.org:act:DB802B45-E18D-4F55-9D23-5401660DD94E

http://species-id.net/wiki/Leptoseris_troglodyta

**Figure 1.**
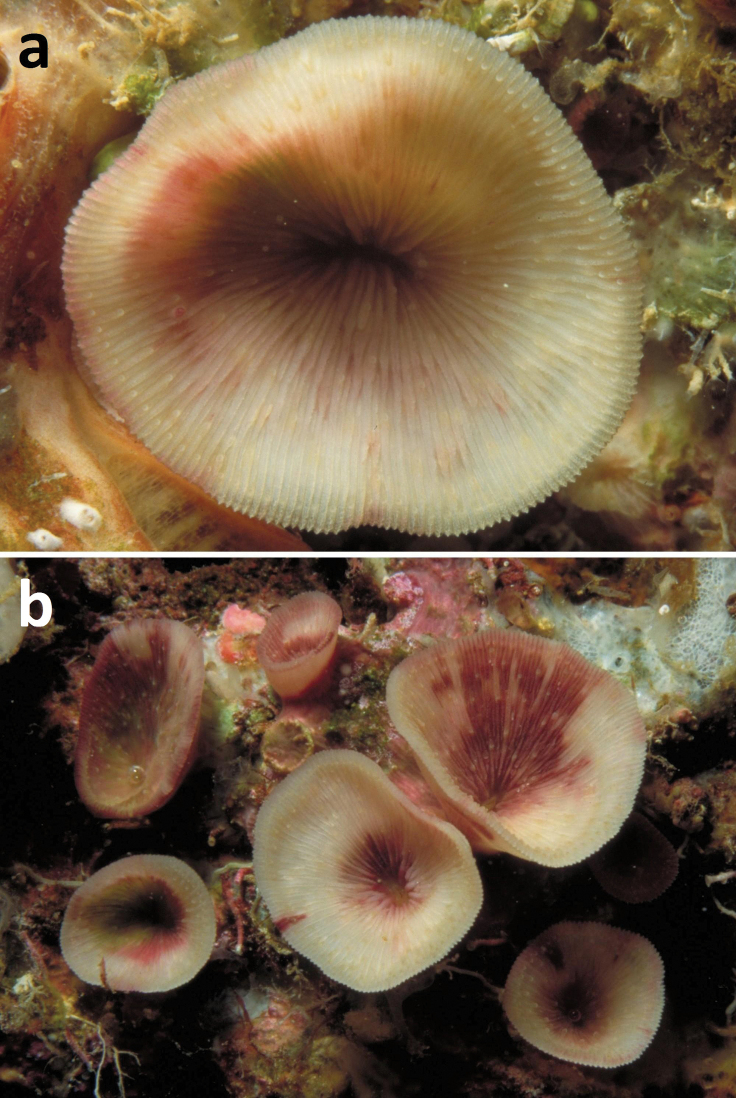
Living specimens of *Leptoseris troglodyta* sp. n. **a** Philippines, Cebu Strait, W of Bohol, NW of Cabilao Island, 10–30 m depth (7 November 1999) **b** Indonesia, NE Kalimantan, Berau Islands, S of Derawan Island, 7–10 m depth (4 October 2003).

Figures 1 [Fig F2] [Fig F3] [Fig F4] [Fig F5] [Fig F6] [Fig F7] –8

Leptoseris sp. Hoeksemaand Van Ofwegen 2004.

##### Type material.

**Figure 2. F2:**
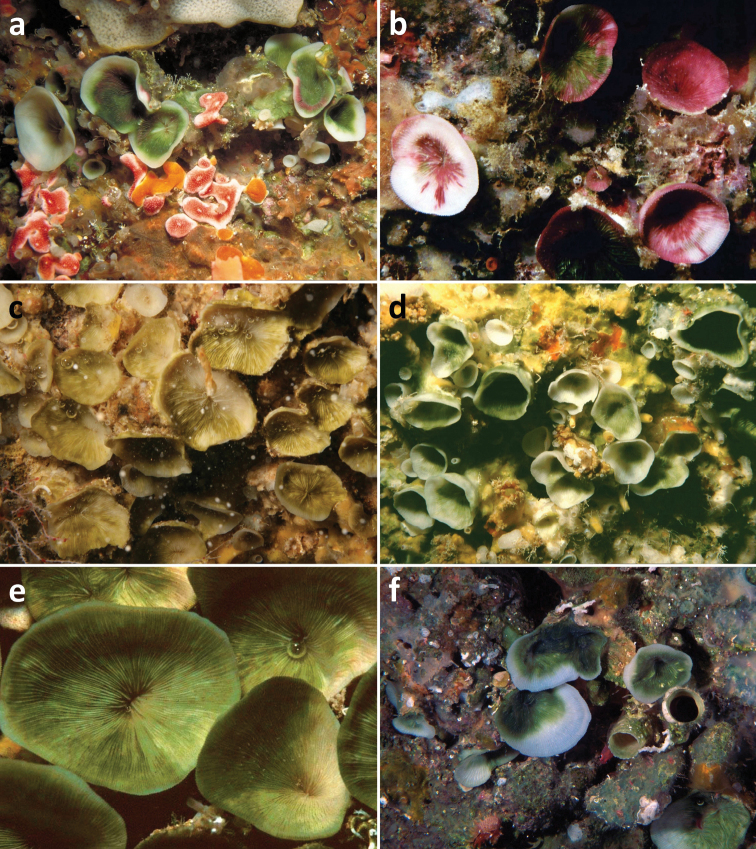
Living specimens of *Leptoseris troglodyta* sp. n. **a** Philippines, Cebu Strait, W of Bohol, NW of Cabilao Island, 10–30 m depth (7 November 1999) **b** Indonesia, Tukang Besi Islands (Wakatobi), Binongko, 20 m depth (10 May 2003) **c** Indonesia, North Sulawesi, S of Bunaken Island, 17 m depth (19 December 2008; photo B.T. Reijnen) **d** Palau, W of Ulong Island (Rattakadokoru Island), W off barrier reef, 32 m depth (28 July 2002) **e** Papua New Guinea, Misima Island, 6–10 m depth (31 May 1998; photo G. Paulay) **f** Guam, Blue Hole, 35 m depth (1 June 2000; photo G. Paulay).

**Figure 3. F3:**
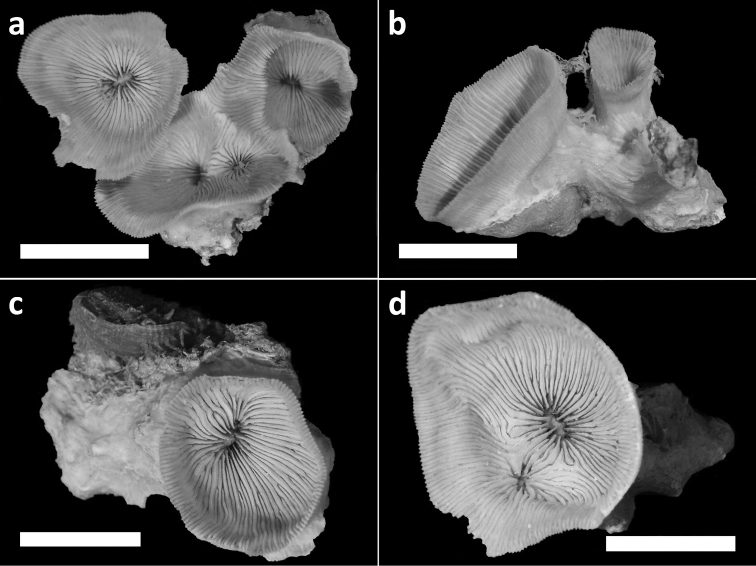
Holotype (RMNH Coel. 40138) and three paratypes (RMNH Coel. 40139) of *Leptoseris troglodyta* sp. n.from Palau. Scale bars: 1 cm. **a** Holotype consists of four calices: one (most left) has fused mid-height its calyx with two totally fused calices (centre), while another (right) has fused only with its corallum margin to those at the centre and the rest of its calyx has remained separate **b** Paratype: two separate calyces **c** Paratype: single calyx **d** Paratype: two fused calices. Scale bars: 1 cm.

Holotype. RMNH Coel. 40138 (1 specimen, dry, Figure 3a), **Palau**, W of Ulong Island (Rattakadokoru Island), W off barrier reef, 07°18'40"N, 134°13'30"E, Tsey’s tunnel ceiling at 32 m depth, 28 July 2002, coll. B.W. Hoeksema. Paratypes: RMNH Coel. 40139 (15 specimens, dry), same collection data (Figures 2d, 3b–d).

##### Other material examined.

**Figure 4. F4:**
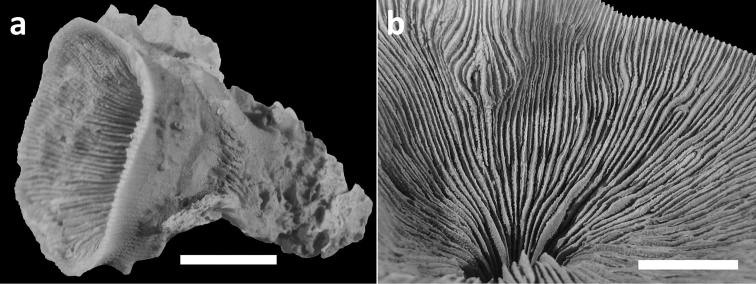
Specimens of *Leptoseris troglodyta* sp. n.from Indonesia, Wakatobi (RMNH Coel. 40152) **a** Single specimen from the side showing cup-shaped calyx shape and costae with fine granular spines (scale bar: 1 cm) **b** Close-up of large calyx showing septa with wide menianes along their sides (see Figure 5; scale bar: 5 mm).

**Figure 5. F5:**
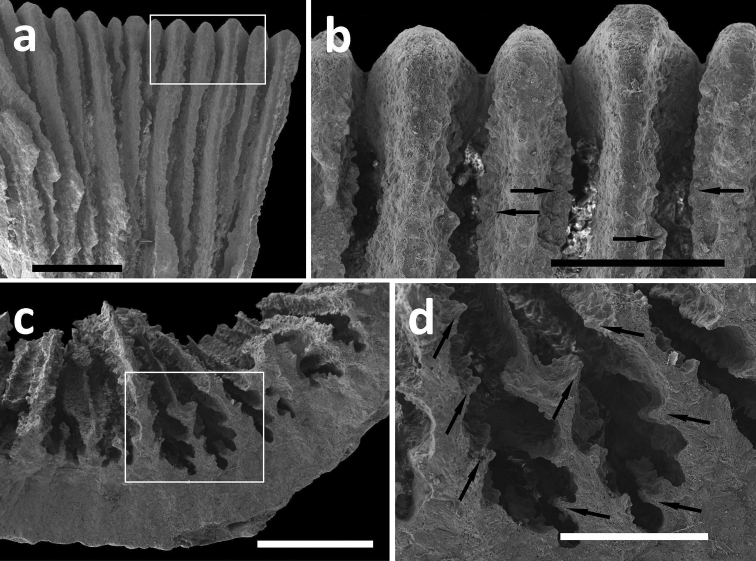
SEM photographs of *Leptoseris troglodyta* sp. n. from Wakatobi, Indonesia (RMNH Coel. 40152). **a** Upper side of septa showing wide menianes (scale bar: 1 mm; insert: Figure 5b) **b** Close-up (insert) of Figure 5a (arrows: menianes; scale bar: 0.5 mm) **c** Cross-section of septa showing multiple menianes along their sides (scale bar: 0.5 mm; insert: Figure 5d) **d** Close-up (insert) of Figure 5c (arrows: menianes; scale bar: 0.5 mm).

**Figure 6. F6:**
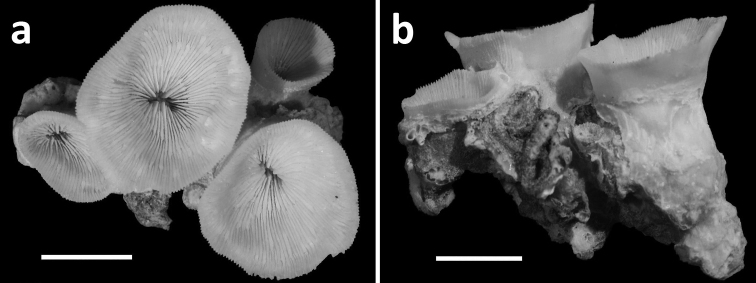
Specimens of *Leptoseris troglodyta* sp. n. from the Philippines, Cebu, Mactan Island, (RMNH Coel. 40151). Scale bars: 1 cm. **a** Upper side of a specimen showing four separate calices **b** Three calices that are partly fused at their sides.

**Figure 7. F7:**
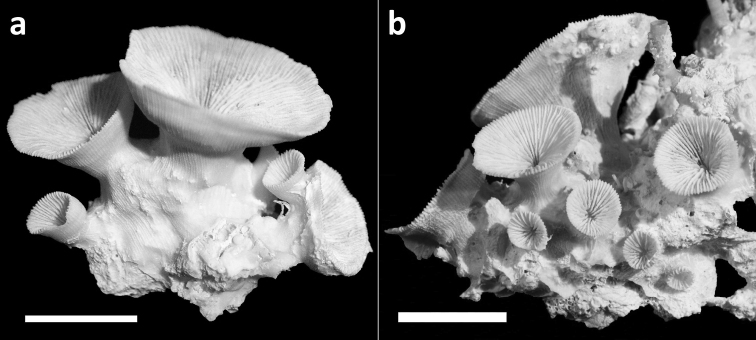
Specimens of *Leptoseris troglodyta* sp. n.from the Philippines, W of Bohol, NW side of Cabilao Island (RMNH Coel 24195). Scale bars: 1 cm. **a** Cluster of calices formed by extra-calicular budding showing costae with small granular spines **b** Idem.

**Figure 8.**
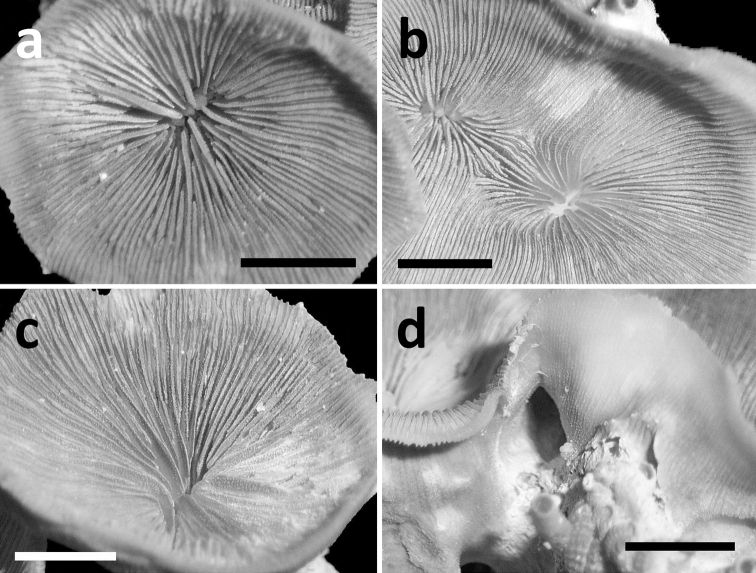
Specimens of *Leptoseris troglodyta* sp. n. from the Philippines, W of Bohol, NW side of Cabilao Island (RMNH Coel 24195). Scale bars: 5 mm **a** Single calyx from above, showing nearly solid columella in the fossa **b** Two totally fused monocentric calices showing, each with its own fossa **c** Upper side of calyx showing clearly visible granulation on septal sides **d** Calyx showing solid corallum wall covered by costae with fine granular spines.

**Philippines.** RMNH Coel. 24187 (3 specimens, dry), RMNH Coel 24195 (36 specimens, dry), Cebu Strait, W of Bohol, NW side of Cabilao Island, 09°53'12"N, 123°45'32"E, vertical wall with caves,10–30 m depth, 7 and 17 November 1999, coll. B.W. Hoeksema (Figures 1a, 2a, 7, 8). RMNH Coel. 40151 (2 specimens, dry), Philippines, Cebu, Mactan Island, Lapu-Lapu City, Marigondon Cave, 10°15'33"N, 123°59'07"E, ceiling of cave at 25-30 m depth, May 1981, coll. M.B. Best (Figure 6). **Indonesia.** RMNH Coel. 40152 (7 specimens, dry), Tukang Besi Islands (Wakatobi), Binongko, SW Bay, 05°59'47"S, 124°02'55"E, cave in steep wall 20 m depth, 10 May 2003, coll. B.W. Hoeksema (Figures 2b, 4–5). RMNH Coel. 40150 (25 specimens, ethanol), Tukang Besi Islands (Wakatobi), NE Hoga Island, 05°28'S, 123°47'E, cave in steep reef slope 5 m depth, 12 July 2011, coll. B.W. Hoeksema. RMNH Coel. 40137 (2 specimens, ethanol), North Sulawesi, South of Bunaken Island, Alung Banua village, 01°37'07"N, 124°45'30"E, tunnel in reef wall 17 m depth, 19 December 2008, coll. B.T. Reijnen and S.E.T van der Meij (Figure 2c).

##### Additional photographic records:

**Indonesia**, NE Kalimantan, Berau Islands, S of Derawan Island, jetty Derawan Dive Resort, 02°17'03"N, 118°14'49"E, ceiling of caves, 7–10 m deep (4 October 2003; Figure 1b). **Papua New Guinea**, Off SE point, Misima Island, Pt. Ebola, 6–10 m depth (31 May 1998; Figure 2e). **Guam**,Blue Hole, 35 m depth (1 June 2000; Figure 2f).

##### Description.

Corallum attached and solitary or colonial by budding from basal coenosteum (Figures 3–4, 6–8). Calices predominantly monocentric, very thin, cup-shaped to foliaceous, height < 15 mm, outline irregularly circular, Ø < 30 mm (Figures 1–4, 6–8). They are usually separate from each other above the interconnecting basal plate (Figures 3b, 6, 7), but can also be fused at their margins or lateral sides (Figures 3a, 8b). Corallum wall massive. Costae equal and well defined, with small spiny protuberances (Figures 4a, 7a, 8d). Septa approximately equal in size, with smooth upper edges and parallel ridges (menianes) on their sides (Figures 3, 5, 8). Septal sides may show evenly distributed granulations where menianes are absent (Figures 8b–c). Columella nearly solid (Figures 3a, 3c–d, 8a–b). Living animals azooxanthellate; corals are white, or partly green or red (Figures 1–2), owing to the presence of endolithic algae (Kühl and Polerecky 2008).

##### Diagnosis.

Corals cave-dwelling, azooxanthellate. Calices small, cup-shaped, monocentric or fused, forming buds at basal coenosteum.

##### Etymology.

The epithet *troglodyta* (noun) means cave dweller in Latin, derived from ancient Greek for “one who dwells in holes”.

##### Distribution.

**Figure 9.**
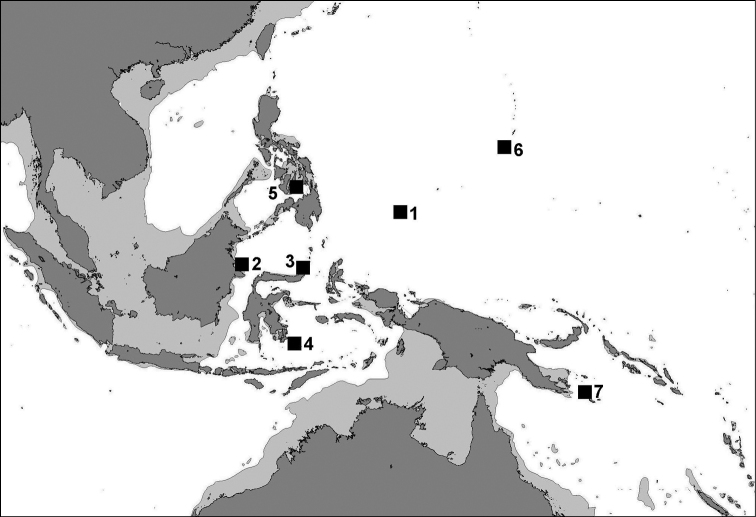
Distribution map of*Leptoseris troglodyta* sp. n. showing records at (**1**) Palau, (**2**) East Kalimantan, (**3**) North Sulawesi, (**4**) Wakatobi, (**5**) Bohol, (**6**) Guam, (**7**), eastern Papua New Guinea.

Records are from coral reefs, usually in areas with limestone outcrops: Indonesia (East Kalimantan, North Sulawesi, Southeast Sulawesi), the central Philippines (Cebu, Bohol), Palau, eastern Papua New Guinea, and the Marianas (Guam) (Figure 9).

## Discussion

### Systematics

*Leptoseris troglodyta* sp. n. has a habitat and growth form unlike any other known *Leptoseris*; its corals are cavernicolous and azooxanthellate, and have small, monocentric calices that may multiply by extramural budding and fuse. Other *Leptoseris* species are polycentric by circumoral and marginal budding (Dinesen 1980, Veron and Pichon 1980, Veron 2000, Licuanan and Aliño 2009). All congeneric species are larger, which may be related to their symbiosis with zooxanthellae. Polycentric species are usually bigger than close relatives with monocentric calices, as demonstrated for mushroom corals (Hoeksema 1991b, Gittenberger et al. 2011).

The new species lacks pigments of its own, like many cavernicolous (= troglobiotic) animals. Although there are no zooxanthellae in its soft tissue, it usually contains green or red shade-adapted endolithic cyanobacteria imbedded in the skeleton, which have also been reported from *Leptoseris fragilis* Milne-Edwards and Haime, 1849 (Schlichter et al. 1997).

Dinesen (1983) mentions the occurrence of “numerous *Leptoseris* cf. *scabra* (G. Hodgson, M. Ross, pers. comm.)”, which were observed in 1981 on the ceiling inside the large Marigondon Cave (Mactan Island, Cebu), the Philippines. They were found “further back in the cave, in gloomier conditions” than the cave entrance at ca 30 m depth. It is likely that this record pertains to specimens of the new species. Two corals of the present study were collected at that site in 1981 and were available for study in the RMNH collection in Leiden. These specimens confirm that the new species was present at that locality at that time. Museum collections may indeed help to retrieve information on the earlier occurrence of coral species that have not been recorded before (Van der Meij et al. 2010, Hoeksema et al. 2011, Van der Meij and Visser 2011). However, without field observations it would not have been possible to know that the new species lacks zooxanthellae.

Despite its unique small monocentric corallites and lack of zooxanthellae, the new species is classified with *Leptoseris* because of similarly shaped septa and costae, and by its thin, cup-shaped or foliaceous coralla somewhat resembling those of *Leptoseris fragilis*. The latter species features corals that can be monocentric, but its calices grow larger (Ø > 50 mm) and may eventually form secondary stomata by intracalicular, circumoral budding. Other extinct agariciid genera predominantly consist of encrusting and massive, polystomatous species (Wells 1954, Veron 2000).

Support from molecular analyses (Stefani et al. in prep) would be needed to justify the position of the new species in a separate genus instead of *Leptoseris*. In that case, it could be appropriate to classify it with *Trochoseris* Milne Edwards & Haime, 1849, an extinct genus (Mid Cretaceous – Oligocene) consisting of corals that are solitary, attached and turbinate or trochoid (Wells 1956). However, molecular analyses cannot support such a transition because no live material is available of this genus.

The corals have a basic growth form like that of *Cladopsammia gracilis* (Milne Edwards and Haime 1848) (Dendrophylliidae), i.e., by extramural budding from the basal coenosteum (see Cairns 1991). They are not distinctly reptoid as described for some *Rhizopsammia* species of the same family (Cairns and Zibrowius 1997) because there are no clear basal costate stolons involved. Extramural budding is also shown by fossils of the extinct genus *Brachyphyllia* Reuss, 1854 (Agariciidae), which has thick, low plocoid corallites (Wells 1956) instead of the thin cup-shaped corallites shown by the new species. Compared to the extant solitary agariciid deep-water species *Dactylotrochus cervicornis* (see Wells 1954, Cairns 1999, Kitahara et al. 2012, Cairns and Kitahara 2012), *Leptoseris troglodyta* differs by the capacity to become colonial and by having a circular corallum outline instead of a periphery with thecal extensions. Only a few specimens of *Dactylotrochus cervicornis* are known to show an ‘aberrant’ tendency for coloniality (Kitahara et al. 2012).

### Evolution of symbiosis with zooxanthellae

*Leptoseris troglodyta* is the first extant shallow-water agariciid known known to be reef-dwelling and azooxanthellate. The extinct agariciid genera, *Trochoseris* Milne Edwards & Haime, 1849 and *Vaughanoseris* Wells, 1934, also consists of monocentric species; the first being attached and turbinate or trochoid, and the second being free-living and discoid (Wells 1956). According to Kitahara et al. (2012) they could have been azooxanthellate because the extant *Dactylotrochus cervicornis*, which shows a basal position in the phylogeny reconstruction of the Agariciidae, is considered both azooxanthellate and monocentric. However, the phylogenetic positions of the two extinct taxa are unknown and although they share a (supposedly plesiosmorph) solitary growth form with *Dactylotrochus*, this does not necessarily imply that they are phylogenetically closely related to each other. For example, several phylogenetic lineages within the reef-dwelling mushroom coral family Fungiidae show an evolution from monocentric to polycentric zooxanthellate corals, implying that not all monocentric fungiid species are directly related to each other (Hoeksema 1989, 1991b, Gittenberger et al. 2011, Benzoni et al. 2012).

Coincidently, the Fungiidae show many examples of solitary free-living species that resemble the extinct *Vaughanoseris*. So, regarding their lifestyle, *Vaughanoseris* species could be reef-dwelling and zooxanthellate as well. Moreover, the attached, monocentric dendrophylliid *Balanophyllia europaea* (Risso, 1826) is an example of a single zooxanthellate species (Schuhmacher and Zibrowius 1985) among a majority of congeners without zooaxanthellae (Cairns et al. 1999). Its growth form resembles that of *Trochoseris*, for which the absence of zooxanthellae may therefore perhaps be less certain. Nevertheless, a solitary growth form, an old fossil record, and a possible ancestral position in the phylogeny may not be sufficient to predict whether an extinct coral taxon may have been azooxanthellate. Its habitat (especially depth) may be more indicative, especially if species were shallow reef-dwellers.

The deep-water species *Dactylotrochus cervicornis* and the cave-dweller *Leptoseris troglodyta* posses parallel ridges on the sides of their septa, which are called menianes (Kitahara et al. 2010, 2012). These are probably the same structures (compare Figure 5, Kitahara et al. 2012 figure 2, and Kahng et al. 2012 figure 9) that help zooxanthellate *Leptoseris* corals to absorb sunlight more efficiently at greater depths (Kahng et al. 2012). The bathymetric records of *Dactylotrochus cervicornis* (Kitahara et al. 2012) partly coincide with depth ranges of zooxanthellate *Leptoseris* species (see Dinesen et al. 2012). Depth may therefore not always be indicative for the absence of zooxanthellae.

All live specimens of *Leptoseris troglodyta*, which were observed in their habitat (Figures 1–2), were clearly azooxanthellate by lacking a brown colour, like completely bleached corals (Hoeksema 1991a, Hoeksema and Matthews 2011). In the case of dredged deep sea corals in collections, which are either dry or being preserved in ethanol, pigments of any present zooaxanthellae might have dissolved, which makes it difficult to see whether they were present. If *Dactylotrochus cervicornis* corals lack zooxanthellae, like those of *Leptoseris troglodyta*, the presence of menianes at least suggests that their ancestors might have been zooxanthellate and that the loss of zooxanthellae may be an evolutionary novelty related to life in deep water or in caves. Consequently, ancestral agariciids, along with *Trochoseris* and *Vaughanoseris*, were perhaps also zooxanthellate like many modern monocentric zooxanthellate reef corals in illuminated habitats. On the other hand, a preceding presence of menianes in agariciid corals may also have facilitated the development of symbiosis with zooxanthellae, which implies that early agariciids may have been azooxanthellate as suggested by Kitahara et al. (2012), presenting a “chicken or the egg” causality dilemma.

The deep-water species *Dactylotrochus cervicornis* and the cave-dweller *Leptoseris troglodyta* both live in dark environments. The latter has been observed to be azooxanthellate in caves at various localities. *Dactylotrochus cervicornis* specimens may have to be examined for the absence of zooxanthellae to be sure that this species is always azooxanthellate. If so, its menianes have no use in connection to light absorption, like in *Leptoseris troglodyta*. *Leptoseris troglodyta* shows that the evolutionary relation between scleractinian reef corals and their algal symbionts is not fixed and that it may be difficult to deduct such a relation based on coral growth forms and their possible position in phylogeny reconstructions, especially if no molecular data can be obtained, as is the case for fossil corals. According to a recent molecular study, coloniality may have become lost at least six times and symbiosis with zooxanthellae at least three times in the phylogeny of the Scleractinia (Barbeitos et al. 2010). However, these numbers are based on a subset of species and may have to be revised if additional species are involved (see Gittenberger et al. 2011).

### Eco-morphological considerations

*Leptoseris troglodyta* sp. n. is the first reef-dwelling agariciid coral without zooxanthellae. As a small cave-dwelling species it can live without sunlight. It has not been observed to co-occur with any zooxanthellate scleractinians (only azooxanthellate species), although in small and poorly illuminated cavities a variety of zooxanthellate scleractinians can be discerned (Dinesen 1983). Other *Leptoseris* species are zooxanthellate and most of them are able to live on deep, poorly illuminated reefs or rocky substrates. Therefore, from an evolutionary perspective, the new species may have lost the capacity to live in symbiosis with zooxanthellae and it may have obtained a smaller corallum size (dwarfism). By their small and thin corolla the corals have little weight. Consequently, with their wide basal plate they may not easily break off from their substrate, which consist of porous limestone cave ceilings where settlement space is limited. Without zooxanthellae, they may not easily reach large sizes, whatsoever. Owing to its modular growth form a *Leptoseris troglodyta* coral may risk losing a few expendable calices while other *Leptoseris* corals may harm and lose their entire corallum in unfavourable conditions. Other examples of zooxanthellae loss in relation to a cavernicolous lifestyle are so far unknown among scleractinian families. However, among shallow-water brachycnemic zoanthids an undescribed cave-dwelling *Palythoa* species has been recorded that also lacks zooxanthellae, while its congeners are known to be zooxanthellate (Reimer 2010).

## Supplementary Material

XML Treatment for Leptoseris troglodyta
